# Health Management in Italian Prisons during COVID-19 Outbreak: A Focus on the Second and Third Wave

**DOI:** 10.3390/healthcare10020282

**Published:** 2022-01-31

**Authors:** Raimondo Vella, Gabriele Giuga, Giorgia Piizzi, Danilo Alunni Fegatelli, Giulia Petroni, Alessandro Mauro Tavone, Saverio Potenza, Andrea Cammarano, Gabriele Mandarelli, Gian Luca Marella

**Affiliations:** 1 Section of Legal Medicine, Department of Biomedicine and Prevention, University of Rome Tor Vergata, 00133 Rome, Italy; giugagabriele@gmail.com (G.G.); giorgia.piizzi@gmail.com (G.P.); giulia.petroni17@gmail.com (G.P.); am.tavone@yahoo.it (A.M.T.); potenza@med.uniroma2.it (S.P.); andrea.cammarano@uniroma2.it (A.C.); 2 Department of Public Health and Infectious Diseases, Sapienza University of Rome, 00185 Rome, Italy; danilo.alunnifegatelli@uniroma1.it; 3 Interdisciplinary Department of Medicine, University of Bari Aldo Moro, 7024 Bari, Italy; gabriele.mandarelli@uniba.it; 4 Forensic Pathology Section, Department of Surgical Sciences, University of Rome Tor Vergata, 00133 Rome, Italy; glmarella@gmail.com

**Keywords:** prison healthcare systems and governance, prison environment, inmates and infectious diseases, COVID-19 and prison, compatibility of the prisoner’s health conditions with imprisonment, prison workers and their protection, penitentiary medicine, clinical risk management in penitentiary medicine

## Abstract

The SARS-CoV-2 spread is a threatening and challenging issue for correctional systems worldwide because of many factors, particularly overcrowding and of the intrinsic characteristics of the population. The prevention measures adopted by the Italian Government were aimed to protect and preserve both inmates’ and prison workers’ health. The present study aimed to evaluate the efficacy of the adopted strategies. Methods: Data regarding Italian prisons’ occupation and prisoners’ population from January 2019 to June 2021, as well as the cumulative weekly increase of confirmed cases and the number of doses of vaccine administered among the population of inmates, the prison workers, and Italian population from November 2020 to the end of June 2021, were collected. Results: Prisons’ occupation dropped from 120% to 106% after the beginning of the pandemics. The confirmed cases between inmates were consistently lower than among the Italian population and prison workers. A time-series chart showed a time lag of one week between the peaks of the different population. Conclusions: The containing strategies adopted by the Italian correctional system have proved their effectiveness in terms of the prevention and protection of both inmate and staff health.

## 1. Introduction

The SARS-CoV-2 epidemic was first detected in China and rapidly spread around the world until, on March 12, 2020, a pandemic was declared [1].

SARS-CoV-2 transmission, as a respiratory-borne illness, is dependent on respiratory contact between individuals, therefore detention settings are extremely susceptible to its rapid and disastrous spread, as documented for other infectious diseases by the historical spread of influenza, tuberculosis, and other respiratory pathogens [2,3].

The progression of the epidemic, as well as the unexpected and unforeseen crisis that it has induced, forced political and institutional entities to modulate their communicational and operative approach. Within this framework, the social and political systems have set their action and communication strategies as in a “combat or a war situation” [4].

WHO/Europe has therefore published the “Preparedness, prevention and control of COVID-19 in prisons and other places of detention” guidelines to provide useful information for prison staff and health care providers and professionals. The document provides advice on the prevention and management of a potential epidemic outbreak, paying particular attention to respect for human rights, and highlights the importance of adapting the operative approach to the local context [5].

The Italian Government, because Italy was one of the first countries to be strongly affected by this emergency, was forced to adopt new and unprecedented measures to contain the spread of SARS-CoV-2 infection. Many measures and strategies were also specifically thought of for prisons and considered the close link between the community, prisoners, and prison employees. Few recent studies have analyzed the impact of SARS-CoV-2 and COVID-19 in the jails of several Regions of the Country and focused on the adopted measures, including the commutation of the sentences to home detention and the implementation of strict hygiene and case-management protocols [6,7,8,9,10,11].

A study that focused on all Italian prisons during the so called “first wave” of the pandemic, i.e., from March to May 2020, demonstrated the efficacy of the adopted measures in the prevention of the spread of the epidemic and particularly in the reduction of mortality [12].

The heterogeneous distribution of the COVID-19 outbreak in Italy during the first wave could also have affected these results. In particular, the spread of the epidemics was earlier in Northern Regions than in Central and in Southern ones [13].

Unfortunately, the global and Italian epidemiological situation has progressively worsened in the following months, spreading homogeneously throughout Italy, and highlighting the critical points and the strengths of Italian local and regional policies and prevention strategies [14].

The aim of the present retrospective study was to evaluate the spread of the virus and the efficacy of the measures adopted in Italian prisons during the period from November 2020 to July 2021 and to compare the evolution of the pandemic between the Italian population and the Italian correctional system.

Within this framework, the current analysis tries to describe and analyze the outcome of the Italian strategy not only in delaying the spread of the SARS-CoV-2 virus within prison but also in keeping the prevalence of COVID-19 consistently below 2%.

## 2. Materials and Methods

We systematically collected the monthly reports about prison occupation from the statistical section [15] of the Italian Ministry of Justice from January 2019 to June 2021. The reports contained raw data about prisons’ occupation (i.e., number of prisoners); the total regular capacity and its distribution per prison; and the inmates’ distribution per prison, sex, and nationality (Italian or foreigner). Semestral data on the inmates’ distribution per age and remaining sentence for the period January 2019 to June 2021 were also collected.

The data of cumulative weekly increase of confirmed cases among the population of inmates, the prison police, and the administrative and management staff, as well as the number of doses of vaccine administered, from the second half of November 2020 to the end of June 2021 were collected from the dedicated section of Italian Ministry of Justice’s website [16]. Data regarding the Italian population for the same time frame were collected from the statistical section of the website of the Italian Ministry of Health [17,18].

A time-series chart was used to better examine the data. Cross correlation analysis, which is a measure of similarity of two series, was used to examine the time lags between the time series of the confirmed cases among the prisoners and employees (police staff and administrative staff), compared to the overall Italian population.

All collected data were exported into a Microsoft Excel spreadsheet. All statistical analyses were performed using Microsoft Excel (Microsoft Corporation, Redmond, WA, USA) and R version 4.0.4 (R Core Team 2016, Vienna, Austria).

## 3. Results

### 3.1. Prisons’ Population and Occupation

The prison population showed no statistically significative heterogeneity regarding its distribution per age, sex, remaining sentence, and nationality in the comparison of the semestral reports.

At the beginning of the period of observation, Italian prisons had a total regular capacity of 50,550 (end of January 2019). The maximum capacity was 50,931 places (February 2020), and the minimum one was 50,438 (April 2019).

**Figure 1:**
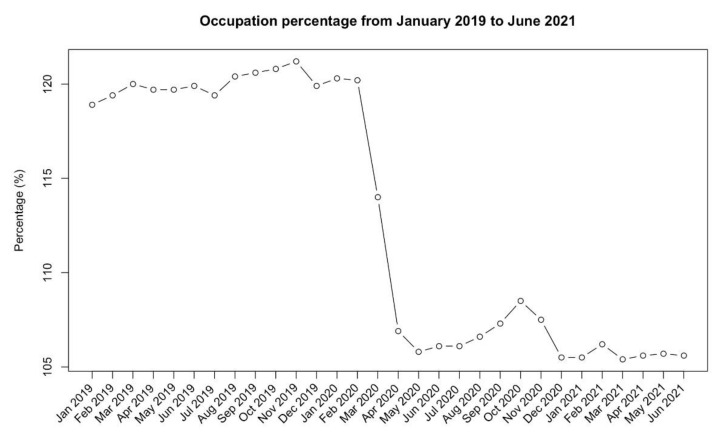
Occupation percentage in Italian prisons from January 2019 to June 2021.

The number of prisoned varied from 60,125 (end of January 2019) to 53,637 (end of July 2021). All the data about prisons’ capacity and the number of prisoners at the end of each month are listed in Table 1 and graphically represented in Figure 1.

The mean prisons’ occupation in the pre-pandemic period of observation (from January 2019 to February 2020) was 120.02% (Range 118.94–121.19%) of the regular prison capacity. A sudden drop of the occupation percentage was observed in the time frame between March and April 2020 because of the application of the prevention measures and of the further discussed effect of Law Decree of 17 March 2020.

During this time frame, 7326 prisoners, with specifical eligibility requirements and by virtue of a short remaining sentence, were released to home detention. After that, the mean occupation percentage was 106.9% (range 105.5–106.87%).

### 3.2. The Control of the Epidemic

**Figure 2:**
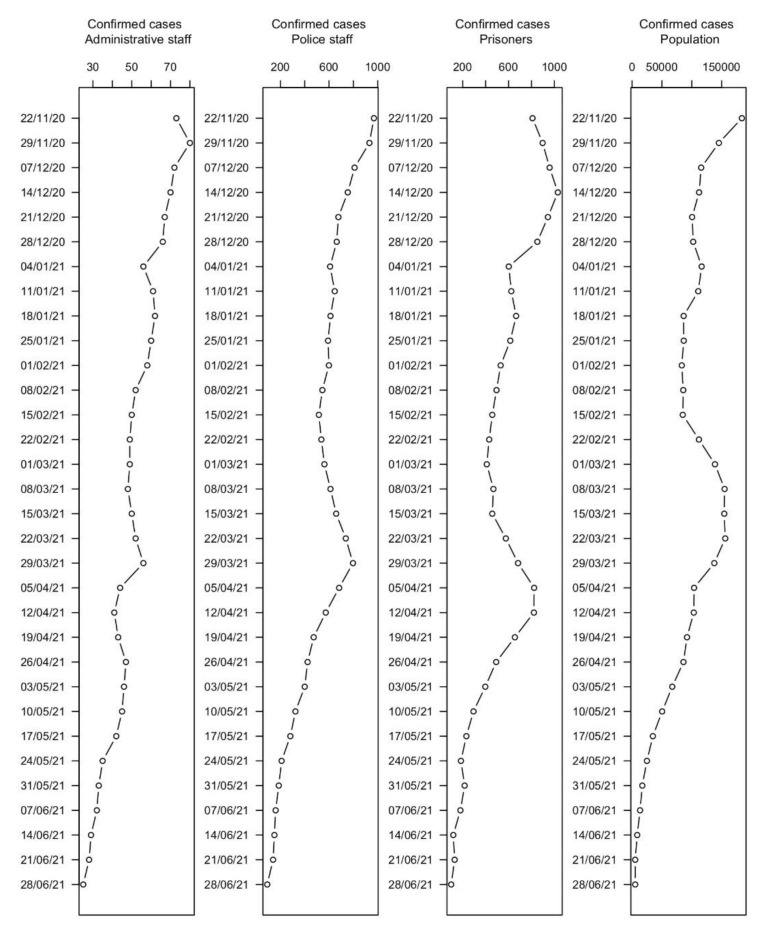
Epidemiological trends of SARS-CoV-2 infection from November 2020 to June 2021: Italian population; prisoners; police staff; and administrative staff.

A time-series chart showed a substantially similar trend in the evolution of the spread of infection, as shown in Figure 2.

Compared to the general population, a similar trend of cases between police officers and administrative staff is observed as they are likely to be a representative sample of the population.

**Figure 3:**
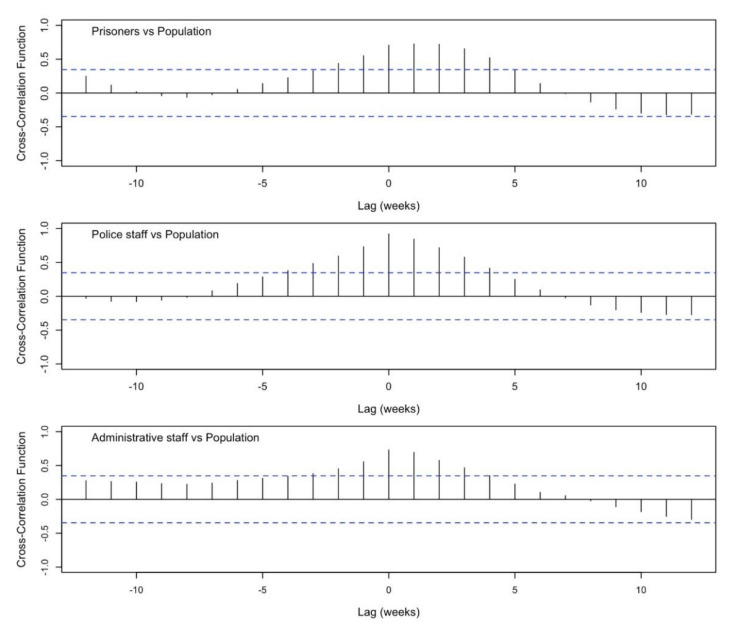
Cross correlation analysis of the time lags between the examined populations.

The cross-correlation plot showed the correlation between two time series at all possible lags in each range. As shown in Figure 3, the peak occurs approximately at lag 1 for the first plot (prisoners vs. population) and at lag 0 for the others (employees vs. population), suggesting a similar trend in the time series. These findings are coherent with the delay of 1–2 weeks in the peak of infections between the inmates and the other populations, suggesting the hypothesis that the infection is spread into the prisons from outside and/or via the police staff.

During the period of observation, the prevalence of SARS-CoV-2 infection among prisoners ranged from 0.19% to 1.94% (mean 1.02%, SD 0.51%). The peak of prevalence was reached at the end of the second week of December 2020. The prevalence ranged from 0.25% to 2.61% (mean 1.43%, SD 0.63%) among police staff and from 0.62% to 1.96% (mean 1.25%, SD 0.34%) among administrative staff. The detailed prevalence percentage of the three populations are detailed in Table 2.

The difference in the spread of the infection may reflect a wider control of the epidemics among the inmates’ population than in the others. This finding is probably attributable to the freedom limitations of the prisoners that could have played a key role in the prevention of the spread, which could not be achieved in a more “open” system.

### 3.3. The Vaccination Campaign

**Figure 4:**
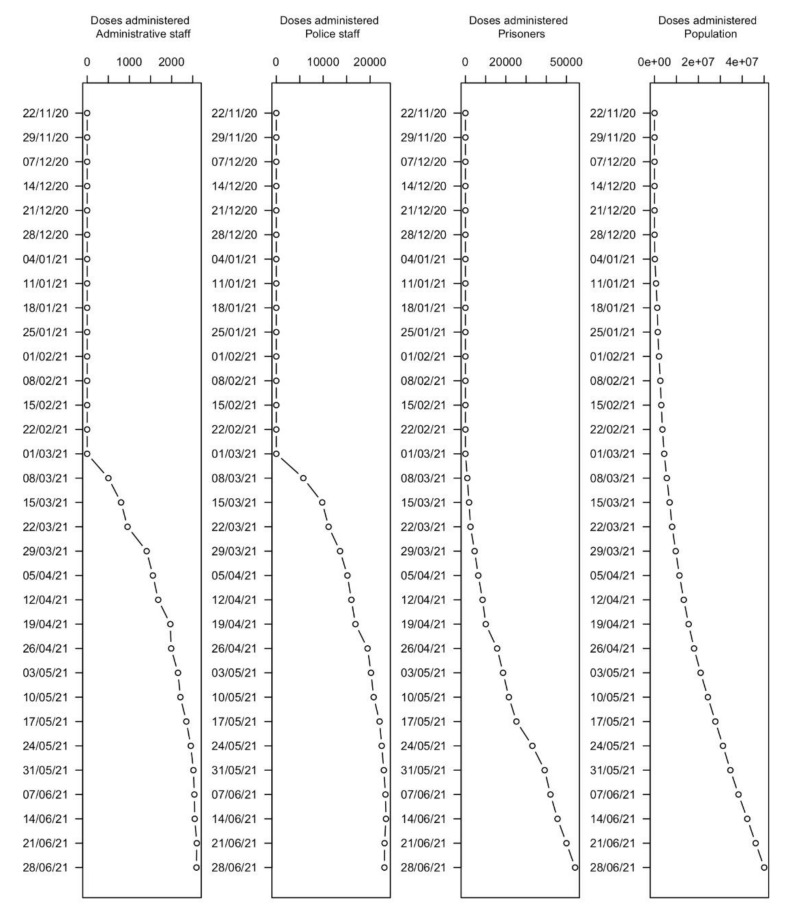
Number of administered doses of vaccine (from top to bottom): Italian population; prisoners; police staff; and administrative staff.

Data regarding vaccination showed a concomitant beginning of the vaccination campaign among prisoners, prison police, and administrative workers, with a delay of 11 weeks if compared to the general population, as synthetically described in Table 3 and graphically represented in Figure 4.

The graphical comparison of the cumulative number of vaccine doses administered showed a similar slope between police and administrative staff. The slopes referring to the Italian population and to prisoners showed a different trend instead. This allows one to infer a different access to vaccination among the overmentioned different groups.

## 4. Discussion

The prevention and control of COVID-19 outbreak in prisons has been assessed as critical issue by many authors [3]. The importance of preserving both inmate and staff health required a series of measures aimed at preventing the spread of COVID-19 but that could have had a strong negative impact on prisoners’ and workers’ mental health [1,19,20].

During a pandemic, one of the main risk factors related to detention is represented by overcrowding. No consensus exists worldwide on how to measure prisoner overcrowding [21]. In this frame, prison cell spatial density (cell floor area per person) is the preferred metric since other measures such as “current prisoner population divided by reported prison capacity” or “number of square meters of the total prison floor area per person” can be manipulated by prison authorities [22].

Most countries worldwide have prison occupancy levels that exceed their officially reported capacity [23], and crowding is linked to adverse health outcomes and transmission of infection. At this regard, nine mediating factors for transmission of infectious disease related to cell spatial density have been detected by authors, including age, education level, pre-existing medical conditions (particularly chronic disorders), hazardous behaviors such as intravenous drug use, environmental ventilation, duration of incarceration, cell allocation, access to prison health service, and prison release to increase spatial separation among remaining prisoners [24].

The problems related to prison overcrowding may imply the adoption of different strategies such as improving hygienic conditions; population screening; case isolation; and prisoner release. Even though mass prisoner release has been considered controversial, many countries have considered it as a possible solution [25].

According to the Italian Ministry of Justice, prison capacity is calculated as 9 m^2^ per single inmate, plus 5 m^2^ for the others in the same cell. The same criterium is adopted to calculate the habitability of private houses [26].

Overcrowding also represents a remarkable question for Italian prisons, where the regular capacity has been highly exceeded, especially in the pre-pandemic period.

People deprived of their liberty, such as people in prisons, are likely to be more vulnerable to various diseases and conditions. Freedom deprivation generally implies that people in prisons and other places of detention live in forced proximity to one another. This condition is likely to result in a heightened risk of person-to-person and droplet transmission of pathogens such as SARS-CoV-2.

In addition to demographic characteristics, people in prisons typically have a greater underlying burden of disease and worse health conditions than the general population, and they frequently face greater exposure to risks such as smoking, poor hygiene and weak immune defense due to stress, and poor nutrition, or prevalence of coexisting diseases, such as bloodborne viruses, tuberculosis, and drug use disorders [2].

This forced countries to adopt individual measures, adapted to the local context.

Since the declaration of the health emergency, with the note of 25 February 2020 [27], the directors of penitentiaries were invited by the Italian Government to define procedures for testing suspected cases of COVID-19 among the prison population, isolating those who tested positive and providing the proper personal protective equipment and protocols for their health care. Specific procedures were instituted to ensure safe entry into the penitentiary for new inmates coming in from the community or from other correctional institutions by creating protected separate pathways.

New detainees were obliged to wash and disinfect their hands and wear a certified medical mask prior to entry, and a new pre-triage filter area system was introduced for prisoners who had access to the outside [7].

The pre-triage system distinguished prisoners in three risk-based categories, providing different instructions for each one, according to Italian Ministry of Health’s provisions: treatment and isolation for symptomatic prisoners (fever of 37.5 °C, sore throat, respiratory difficulty, and flu-like/COVID-19-like symptoms/pneumonia); evaluation for asymptomatic or pauci-symptomatic and negative tested inmates in close contact with a confirmed case; and isolation for 14 days for prisoners who were asymptomatic and tested positive [8].

Apart from this, the Law-Decree n. 18 of 17 March 2020, converted into Law no. 27 of 24 April 2020 [28], introduced provisions that concern prisoners, providing for the possibility for home detention for those who have less than 18 months’ sentence to serve. The measures will be applied by the supervising magistrate, not only at the request of the detainee but also by the public prosecutor or the prison governor. Prisoners with sentences of 7 to 18 months may be able to wear an electronic bracelet to be made available according to a particular distribution program adopted by the prison governor and approved by the head of the public security department. The Decree provided, among other extraordinary measures, specific dispositions for the prison population, with the aim of protecting their health and that of prison staff to contain the spread of the pandemic.

These measures included the suspension of interviews with relatives or other persons, to which sentenced persons, internees, and defendants are entitled, supported by remote communication tools. For this purpose, the prison administration entered into specific agreements with a telephone company to ensure faster and more frequent access of detainees with their families, inviting the prison directors to represent these possibilities to the detained persons. Nevertheless, between 7 and 9 March 2020, violent riots occurred in several Italian prisons. In fact, one of the greatest difficulties during the pandemic emergency was the prohibition of direct communication with family members [19]. This was considered the main factor underlying the inmates’ manifestations of unease, even more than the fear of the virus. The initial deprivation of contact with their families was critical for the well-being of the prisoners, who often openly manifested their unhappiness at times through violent actions.

Further measures adopted were the availability of personal protection equipment to all prison staff, the identification of spaces for isolation and observation of suspected cases, the adoption of swabs, the suspension of interviews with defense lawyers, the suspension of audiences (except for urgent ones to be held electronically), the blocking of transfers and movements of detainees, and the isolation of prison staff in suspected situations and/or those who had tested positive via swabs.

In addition, the Department of Penitentiary Administration (DAP) also adopted measures to contain the risk of individual infection in persons suffering from other pathologies that could in some way have favored the transmission of the virus, extending the possibilities of alternative measures to detention in prison (like house arrest) for this group [12], involving even the groups of drug users and people incarcerated for drug-related crimes [29].

The transfer of prisoners from jail custody to home detention has led to a considerable reduction of the prison population. However, it is impossible to evaluate the final impact of this measure on prisoners’ health, since, as previously described, the selection of the inmates eligible for home detention was based only on elements of “social hazard” of those individuals, e.g., the remaining sentence. The eligibility criteria therefore did not consider eventual pre-existing medical conditions related to the risk of developing severe form of COVID-19. This issue is difficult to solve on the basis of available data since the Italian Minister of Justice released only semestral cumulative data about the demography of the inmates and no further information can be found about the distribution of susceptible medical conditions among the inmates’ population. Individuals more susceptible to severe COVID-19 were practically indistinguishable from the eligible transferable prisoners, and this was confirmed, for example, by the fact that the reduction of prison population was not accompanied by a modification of the prisoners’ demographic characteristics based on their age classes.

In this regard, the protective effect of reduction of prison overcrowding might have had an impact on the control of the epidemic, but its efficacy cannot be considered as independent from the other hygiene and prevention measures.

Nonetheless, lowering prison density may have improved the quality of life in the detention setting.

Particular attention should also be paid to the impact of the vaccination plan. With the advent of available COVID-19 vaccines, by the beginning of December 2020 the Italian Ministry of Health presented to Italian Parliament a strategic plan to implement a vaccination program based on the initial availability of vaccines [30].

This plan was finally adopted with a Decree of the Ministry of health on 2 January 2021. Priority of access to vaccination was initially given to health professionals and fragile population (elderly and heavy impaired people). Other priority risk categories were identified by a further decree of 12 March 2021 [31].

Within this framework, prisoners as well as prison workers were included among the priority targets of vaccination, together with armed forces and public workers, due to the complexity of their population and to the potential risk of infection. This has led to an immediate implementation of the vaccinations among those categories.

Some authors have taken issue with the access to vaccinations as an effective and critical problem in terms of prevention, due to the intrinsic fragility of the inmate population [8]. Despite their priority for vaccination, the inmates seem, however, to have had a slower and more prolonged time of access, if compared to police and prison workers, who belonged to the same risk class.

However, the incarcerated population showed lower infection rates after the implementation of the vaccination plan, if compared to the other populations. Additionally, in this regard, it is particularly difficult to evaluate the impact this measure could have made since it was impossible to estrange it from the other adopted measures and from the freedom limitation itself.

Given the criticalities of the Italian correctional system at the starting point, even if it does not seem possible to establish the exact contribution of each of the above-mentioned aspects or which one may have had a predominant effect, the whole result of the adopted strategies may be considered successful in terms of control of the spread of the infection among inmates’ population compared to the much greater upward trend of the Italian population. Further studies may clarify this.

## 5. Conclusions

After a homogeneous spread of the COVID-19 epidemic during the second and third wave all over the Country, the containing strategies adopted by Italian correctional system have proved their effectiveness in terms of prevention and protection of both inmate and staff health. This success is attributable to many factors such as the reduction of prison overcrowding, the strict application of isolation and case detection protocols and the implementation of vaccinations for both inmates and prison staff. Since prisons cannot be considered a completely ‘closed’ system and are not separated from the community, additional measures should be adopted to prevent COVID-19 spread among prison workers, to consequently protect inmates’ health and improve efficacy.

A major effort should have been made to gain wider access to vaccination among prisoners. Particular attention should be required in the near future on the long term consequences of the application of these measures on prisoners’ health, including mental health and respect for human rights.

## Figures and Tables

**Table 1:** Prisons’ occupation from January 2019 to June 2021.

Month	Prison Regular Capacity	Prisoners	Occupation
January 2019	50,550	60,125	118.94%
February 2019	50,522	60,348	119.45%
March 2019	50,514	60,611	119.99%
April 2019	50,511	60,439	119.66%
May 2019	50,528	60,476	119.69%
June 2019	50,496	60,522	119.86%
July 2019	50,480	60,254	119.36%
August 2019	50,469	60,741	120.35%
September 2019	50,472	60,881	120.62%
October 2019	50,474	60,985	120.82%
November 2019	50,476	61,174	121.19%
December 2019	50,688	60,769	119.89%
January 2020	50,692	60,971	120.28%
February 2020	50,931	61,230	120.22%
March 2020	50,754	57,846	113.97%
April 2020	50,438	53,904	106.87%
May 2020	50,472	53,387	105.78%
June 2020	50,501	53,579	106.09%
July 2020	50,558	53,619	106.05%
August 2020	50,574	53,921	106.62%
September 2020	50,570	54,277	107.33%
October 2020	50,553	54,868	108.54%
November 2020	50,568	54,368	107.51%
December 2020	50,562	53,364	105.54%
January 2021	50,551	53,329	105.50%
February 2021	50,551	53,697	106.22%
March 2021	50,779	53,509	105.38%
April 2021	50,785	53,608	105.56%
May 2021	50,780	53,660	105.67%
June 2021	50,779	53,637	105.63%

**Table 2:** Confirmed cases among prisoners and prison staff.

Week	Prisoners			Police Staff			Administrative Staff		
	*n*	Confirmed Cases		*n*	Confirmed Cases		*n*	Confirmed Cases	
22 November 2020	53,723	809	1.51%	37,153	969	2.61%	4090	73	1.78%
29 November 2020	53,489	897	1.68%	37,153	932	2.51%	4090	80	1.96%
7 December 2020	53,294	958	1.80%	37,153	810	2.18%	4090	72	1.76%
14 December 2020	53,052	1030	1.94%	37,153	754	2.03%	4090	70	1.71%
21 December 2020	52,597	943	1.79%	37,153	677	1.82%	4090	67	1.64%
28 December 2020	51,887	851	1.64%	37,153	663	1.78%	4090	66	1.61%
4 January 2021	52,237	602	1.15%	37,153	609	1.64%	4090	56	1.37%
11 January 2021	52,404	624	1.19%	36,939	647	1.75%	4021	61	1.52%
18 January 2021	52,411	666	1.27%	36,939	612	1.66%	4021	62	1.54%
25 January 2021	52,363	615	1.17%	36,939	592	1.60%	4021	60	1.49%
1 February 2021	52,549	531	1.01%	36,939	599	1.62%	4021	58	1.44%
8 February 2021	52,419	495	0.94%	36,939	545	1.48%	4021	52	1.29%
15 February 2021	52,491	458	0.87%	36,939	516	1.40%	4021	50	1.24%
22 February 2021	52,522	431	0.82%	36,939	537	1.45%	4021	49	1.22%
1 March 2021	52,644	410	0.78%	36,939	562	1.52%	4021	49	1.22%
8 March 2021	52,599	468	0.89%	36,939	612	1.66%	4021	48	1.19%
15 March 2021	52,591	458	0.87%	36,939	659	1.78%	4021	50	1.24%
22 March 2021	52,572	576	1.10%	36,939	738	2.00%	4021	52	1.29%
29 March 2021	52,532	683	1.30%	36,939	797	2.16%	4021	56	1.39%
5 April 2021	52,207	823	1.58%	36,939	683	1.85%	4021	44	1.09%
12 April 2021	52,466	821	1.56%	36,939	573	1.55%	4021	41	1.02%
19 April 2021	52,471	655	1.25%	36,939	474	1.28%	4021	43	1.07%
26 April 2021	52,591	492	0.94%	36,939	424	1.15%	4021	47	1.17%
3 May 2021	52,638	397	0.75%	36,939	400	1.08%	4021	46	1.14%
10 May 2021	52,561	294	0.56%	36,939	324	0.88%	4021	45	1.12%
17 May 2021	52,587	232	0.44%	36,939	282	0.76%	4021	42	1.04%
24 May 2021	52,485	185	0.35%	36,939	210	0.57%	4021	35	0.87%
31 May 2021	52,678	217	0.41%	36,939	187	0.51%	4021	33	0.82%
7 June 2021	52,517	180	0.34%	36,939	161	0.44%	4021	32	0.80%
14 June 2021	52,556	118	0.22%	36,939	111	0.41%	4021	29	0.72%
21 June 2021	52,579	130	0.25%	36,939	140	0.38%	4021	28	0.70%
28 June 2021	52,453	100	0.19%	36,939	92	0.25%	4021	25	0.62%

**Table 3:** Vaccine doses administered among Italian population, prisoners, and prison staff.

Week	Italian Population	Prisoners	Police Staff	Administrative Staff
22 November 2020	0	0	0	0
29 November 2020	0	0	0	0
7 December 2020	0	0	0	0
14 December 2020	0	0	0	0
21 December 2020	0	0	0	0
28 December 2020	7223	0	0	0
4 January 2021	124,806	0	0	0
11 January 2021	674,043	0	0	0
18 January 2021	1,219,290	0	0	0
25 January 2021	1,463,017	0	0	0
1 February 2021	2,024,341	0	0	0
8 February 2021	2,632,032	0	0	0
15 February 2021	3,069,346	0	0	0
22 February 2021	3,595,051	0	0	0
1 March 2021	4,401,107	0	0	0
8 March 2021	5,570,740	927	5764	503
15 March 2021	6,881,713	1799	9797	803
22 March 2021	8,007,921	2500	11,151	956
29 March 2021	9,633,827	4540	13,592	1409
5 April 2021	11,347,927	6356	15,155	1557
12 April 2021	13,325,482	8485	15,998	1683
19 April 2021	15,536,203	10,054	16,869	1970
26 April 2021	18,012,826	15,684	19,451	1990
3 May 2021	21,010,993	18,619	20,178	2151
10 May 2021	24,316,685	21,489	20,758	2208
17 May 2021	27,736,740	25,232	22,011	2349
24 May 2021	31,207,989	33,127	22,464	2454
31 May 2021	34,585,435	39,203	22,918	2516
7 June 2021	38,301,429	42,064	23,266	2539
14 June 2021	42,298,372	45,574	23,370	2547
21 June 2021	46,123,076	50,001	23,072	2597
28 June 2021	50,027,325	54,260	23,041	2589

## Data Availability

All analyzed raw data can be publicly found in the statistical section of Italian Ministry of Justice (https://www.giustizia.it/giustizia/it/mg_1_14.page accessed on 26 October 2021) (https://www.giustizia.it/giustizia/it/mg_2_27.page accessed on 26 October 2021) and of Italian Ministry of Health (https://opendatadpc.maps.arcgis.com/apps/dashboards/b0c68bce2cce478eaac82fe38d4138b1 accessed on 26 October 2021) (https://www.governo.it/it/cscovid19/report-vaccini/ accessed on 26 October 2021).
